# Are mimics monophyletic? The necessity of phylogenetic hypothesis tests in character evolution

**DOI:** 10.1186/1471-2148-10-239

**Published:** 2010-08-03

**Authors:** Jeffrey C Oliver, Kathleen L Prudic

**Affiliations:** 1 Department of Ecology and Evolutionary Biology, Yale University, New Haven, CT 06511, USA

## Abstract

**Background:**

The processes governing the origin and maintenance of mimetic phenotypes can only be understood in a phylogenetic framework. Phylogenetic estimates of evolutionary relationships can provide a context for analyses of character evolution; however, when phylogenetic estimates conflict, rigorous analyses of alternative evolutionary histories are necessary to determine the likelihood of a specific history giving rise to the observed pattern of diversity. The polyphenic butterfly *Limenitis arthemis *provides a case in point. This species is comprised of three lineages, two of which are mimetic and one of which is non-mimetic. Conflicting estimates of the relationships among these three lineages requires direct evaluation of the alternative hypotheses of mimicry evolution.

**Results:**

Using a coalescent framework, we found support for a sister-taxon relationship between the non-mimetic *L. a. arthemis *and the mimetic *L. a. astyanax*, congruent with the previous hypothesis that the non-mimetic form of *L. a. arthemis *was derived from a mimetic ancestor. We found no support for a mimetic clade (*L. a. astyanax *+ *L. a. arizonensis*) despite analyzing numerous models of population structure.

**Conclusions:**

These results provide the foundation for future studies of mimicry, which should integrate phylogenetic and developmental analyses of wing pattern formation. We propose future analyses of character evolution accommodate conflicting phylogenetic estimates by explicitly testing alternative evolutionary hypotheses.

## Background

Batesian mimicry and the conditions favoring its origin and maintenance have provided insight to the process of natural selection. Central to our understanding of Batesian mimicry is the evolutionary fate of mimics in the absence of their model. That is, once a profitable species evolves to mimic an unprofitable Batesian model, what happens in time or space when the model is not present? The Batesian mimic could persist in locations without its model, especially when predation is weak [1,2]. A mimic could also go extinct in these locations due to intense predation [2]. Or the mimic could evolve a new color pattern to mimic another model species or revert back to its ancestral, non-mimetic phenotype [3,4].

Empirical phylogenetic trees are estimations, or hypotheses, of the true evolutionary history of a given group, based on a fit to observed data (morphological characters, DNA sequences, etc.). Such trees can be used as a "best estimate" for studies of character evolution, especially when trees based on different analyses and data converge on the same estimate of evolutionary relationships. But how to proceed with analyses of character evolution when phylogenetic estimates conflict with one another? One approach is to compare trees on the basis of some objective function (number of parsimony steps, likelihood, Bayesian posterior probability, etc.), and simply interpret the tree with the best score as the true evolutionary history. However, because empirical phylogenetic tree estimates do not always reflect true evolutionary history [5,6], inferring a "best estimate" tree does not eliminate the possibility that an alternative evolutionary history gave rise to the observed pattern of character data. By way of analogy, comparing trees based on an objective function alone is similar to comparing the means of two population variables to assess whether the distributions of the variables are different between the two populations. Comparisons between means, and trees in this case, must account for the potential variation in the underlying distributions which gave rise to the observed data.

The necessity of evaluating support for alternative phylogenetic hypotheses has been recognized for some time [7], but has not yet become common practice. Methodological limitations and the stochastic nature of molecular evolution may contribute to misleading phylogenetic estimates [8], so when trees conflict, one must account for the possibility that an alternative phylogeny underlies the history that generated the observed data. However, unlike the analogy with means presented above, evolutionary inferences are constrained to a single observation, so there is no empirical measure of variance in observed data. Parametric bootstrapping [9,10] can be used to simulate expected distributions of data corresponding to specific evolutionary hypotheses (e.g. trees). These distributions are then compared with observed data to assess the relative support for alternative phylogenies. Alternative hypotheses in which the expected distribution does not match observed data are rejected; when observed data fall within the expected distribution, those alternative hypotheses remain plausible, and must be accounted for in hypotheses of character evolution.

The butterfly genus *Limenitis *(Fabricius) (Lepidoptera: Nymphalidae) has long been a model for the study of Batesian mimicry and is an ideal system to employ phylogenetic hypothesis testing. Three of the four North American species include populations which are Batesian mimics of distasteful models [11-14]. *Limenitis arthemis *(Drury) includes two populations, *L. a. astyanax *(Fabricius) and *L. a. arizonensis *Edwards, which are mimics of the distasteful model *Battus philenor *(L.) (Lepidoptera: Papilionidae), and a non-mimetic population, *L. a. arthemis *(Drury), characterized by a disruptive non-mimetic wing pattern [12,15]. The distribution of the phenotypes (mimetic or non-mimetic) is predicted by the distribution of the model species' host plants (*Aristolochia *spp.), which limits the distribution of the model [4]. Although gene flow occurs between the mimetic *L. a. astyanax *and the non-mimetic *L. a. arthemis *[15,16], differing selection pressures in the presence and absence of the model species presumably maintains the polymorphism in wing phenotypes [15]. The origin of the mimetic phenotype, as well as that of the non-mimetic wing pattern of *L. a. arthemis *has elicited recent attention [4,17].

**Figure 1:**
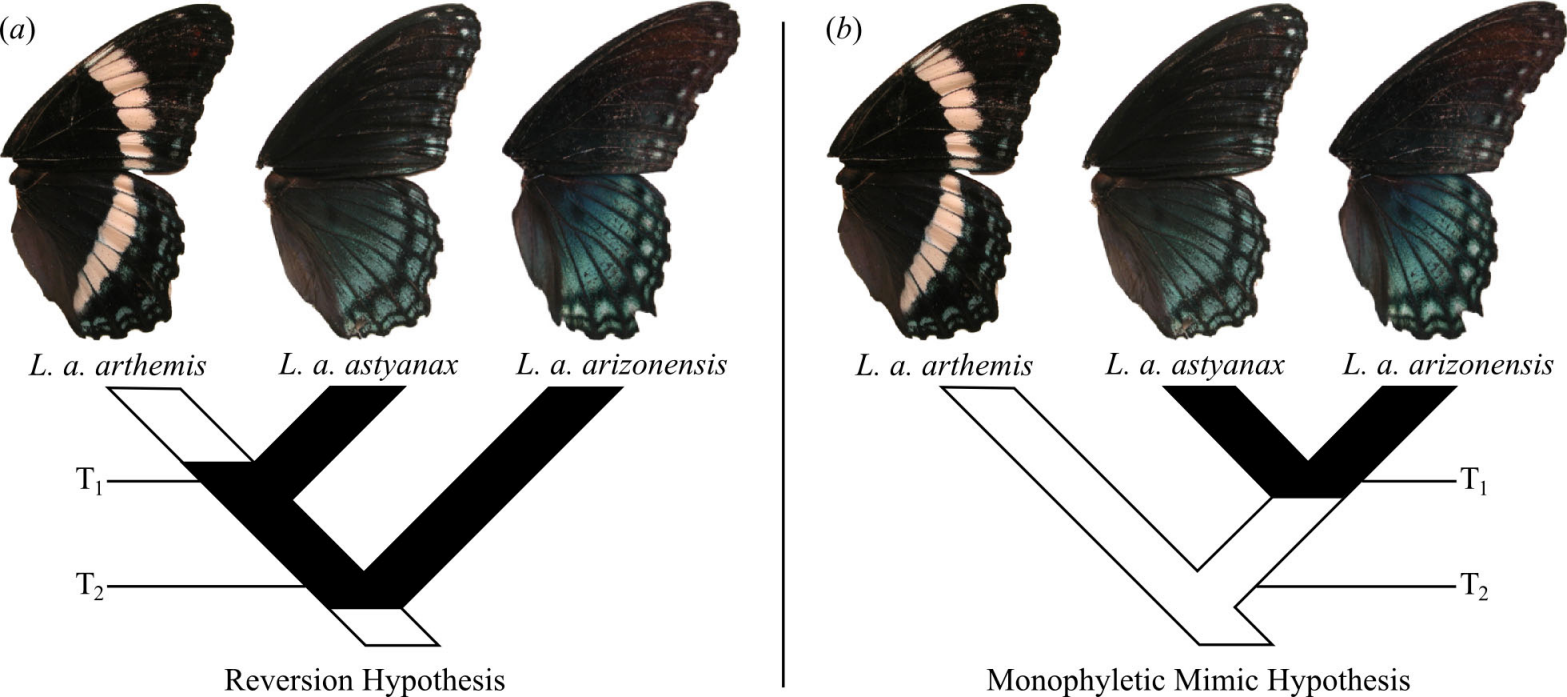
**Two hypotheses of mimicry evolution in *Limenitis arthemis *lineages**. In the reversion hypothesis (a), mimetic *L. a. astyanax *is sister to non-mimetic *L. a. arthemis*. Under this hypothesis, the mimetic phenotype arose in the ancestor to all *L. arthemis *and was subsequently lost in the *L. a. arthemis *lineage. In (a), T_1 _is the divergence time of *L. a. arthemis *and *L. a. astyanax *and T_2 _is the divergence time of *L. a. arizonensis *from the lineage giving rise to *L. a. arthemis *and *L. a. astyanax*. In the monophyletic mimic hypothesis (b), the mimetic lineages *L. a. astyanax *and *L. a. arizonensis *are sister taxa. T_1 _is the divergence time of *L. a. astyanax *and *L. a. arizonensis *and T_2 _is the divergence of *L. a. arthemis *from the lineage giving rise to the mimetic lineages.

Multi-locus DNA sequence estimates of North American *Limenitis *relationships posit that the mimetic *L. a. arizonensis *diverged from a lineage which eventually gave rise to the mimetic *L. a. astyanax *and the non-mimetic *L. a. arthemis *[4,18]. Additionally, explicit hypothesis tests based on mitochondrial DNA sequences rejected the hypothesis that the mimetic lineages *L. a. astyanax *and *L. a. arizonensis *form a clade [4]. The topology of such a relationship suggests that mimicry either evolved once, and was subsequently lost in the lineage ultimately leading to *L. a. arthemis*, or was gained two times, once in *L. a. astyanax *and once in *L. a. arizonensis *[18]. Based on these phylogenetic estimates and tests, along with the biogeography of *L. arthemis *and the model *B. philenor*, Prudic & Oliver [4] advocated the hypothesis that the mimetic phenotype evolved in the ancestral *L. arthemis *lineage and was subsequently lost in *L. a. arthemis *after the divergence of *L. a. arthemis *and *L. a. astyanax *(figure 1a) [18]. A recent phylogeny based on AFLP data challenges this view [17]. Based on a distance analysis of eleven individuals, the authors [17] proposed that the mimetic lineages are sister taxa, and that the mimetic phenotype arose only once in the *L. arthemis *lineage and was not subsequently lost (figure 1b). These two conflicting hypotheses beg the question: which evolutionary process best explains the observed data? Is a loss of mimicry a plausible explanation for the observed wing diversity?

Here we evaluate support for the two hypotheses of wing pattern evolution in *Limenitis arthemis *by performing explicit phylogenetic hypothesis tests. The first hypothesis, the reversion hypothesis (figure 1a) posits that *L. a. astyanax *and *L. a. arthemis *are sister taxa, while the second, the monophyletic mimic hypothesis, asserts that the mimetic *L. a. astyanax *and *L. a. arizonensis *form a clade (figure 1b). We employed a parametric bootstrapping approach, using coalescent simulations of population structure to determine which evolutionary histories would be most likely to produce the observed pattern of genetic diversity. Simulating data under various models based on previous studies [16], we assess the support for the two hypotheses of *L. arthemis *wing pattern evolution. With these results in hand, we discuss the relative likelihood of the gain and loss of mimetic phenotypes in *L. arthemis*. The analyses demonstrate the power of explicit phylogenetic hypothesis tests and provide exciting directions for the future study of mimicry evolution.

## Methods

To test the two hypotheses of wing pattern evolution (figure 1), we used gene tree estimates from eight nuclear loci (see Gene tree sources below) combined with coalescent simulations of gene trees to evaluate support for a variety of models of population structure. We compared the observed value of two statistics (see Model evaluation below), to distributions of these statistics based on simulated gene trees. Correspondence between observed and simulated values of the test statistics were used as measures of support for or against a particular model of evolution.

### Gene tree sources

We included gene tree estimates for eight nuclear loci from previous studies [4,16,18]: four protein-coding loci (*elongation factor 1 alpha *(*EF1α*), *wingless *(*wg*), *kettin*, and *lactate dehydrogenase *(*Ldh*)) and four anonymous loci (*Anon6*, *Anon10*, *Anon15*, and *Anon17*) (GenBank accession numbers available in Additional File 1). We used the consensus of Bayesian MCMC searches for each locus (Additional Files 2 and 3) for calculating observed values of test statistics.

For simulated gene trees, we used coalescent simulations performed in the software package MS [19]. For each simulation replicate, we simulated eight gene trees, with one tree each corresponding to the sampling effort represented by the observed gene trees. That is, for each of eight nuclear loci, we simulated a gene tree with the same number of individuals sampled from each of the six lineages included in this study (table 1).

**Table 1:** Sampling of North American *Limenitis*

Locus	*L. archippus*	*L. a. arizonensis*	*L. a. arthemis*	*L. a. astyanax*	*L. lorquini*	*L. weidemeyerii*
*Anon06*	1	2	11	7	1	1
*Anon10*	1	2	11	8	1	1
*Anon15*	1	1	11	9	0	1
*Anon17*	0	2	13	8	1	1
*EF1a*	22	12	14	16	17	15
*kettin*	1	4	11	9	1	1
*Ldh*	1	3	11	9	1	1
*wg*	7	2	16	4	1	4

Number of individuals sampled from each lineage of North American *Limenitis *for each locus included in this study. Data originally presented in [4,16,18].

### Models of *Limenitis *evolution

We evaluated 15 models of population structure, twelve models corresponding to the hypothesis that the two mimetic lineages, *L. astyanax *and *L. arizonensis*, are monophyletic ('MM' models, figure 1b), and three models corresponding to the reversion hypothesis, in which the mimetic *L. a. astyanax *is most closely related to the non-mimetic *L. a. arthemis *('R' models, figure 1a). For each model being tested, we used parameter estimates from previous analyses and mitochondrial DNA divergences (table 2) (Additional File 2). The MM models included three divergence time estimates for the split of *L. a. arthemis *from the lineage leading to *L. a. astyanax *and *L. a. arizonensis*, each of which included two divergence time estimates of *L. a. astyanax *and *L. a. arizonensis*. These six models were each evaluated under two estimated migration rates, based on previously published analyses [16]. 'Moderate' migration models used the maximum likelihood estimates of population migration rates (measured in number of migrants per generation): 3.2 *L. a. astyanax *to *L. a. arthemis *and 0.14 *L. a. arthemis *to *L. a. astyanax*. 'High' migration models used the maximum of the 90% posterior density intervals: 17.71 *L. a. astyanax *to *L. a. arthemis *and 15.53 *L. a. arthemis *to *L. a. astyanax*. The three R models differed from one another only in their estimated divergence time between *L. a. arthemis *and *L. a. astyanax*. All 15 models had identical divergence time estimates for the three remaining North American *Limenitis *species (Additional Files 2 and 4), and all models included hybridization between *L. a. arthemis *and *L. a. astyanax *beginning 12,000 ybp, following the recession of the Laurentide ice sheet [20]. Additionally, the effective population size of each lineage was 2.5 million individuals, all lineages had two generations per year, and the ancestral lineage that gave rise to the three lineages of *L. arthemis *had an effective population size of 350,000, based on previous estimates [16].

**Table 2:** Parameters used in models of *Limenitis *history

Hypothesis	Migration	Model	T_1_	T_2_
Monophyletic Mimic	Moderate	MM1	117,500	235,000
		MM2	211,500	235,000
		MM3	327,500	655,000
		MM4	589,500	655,000
		MM5	537,000	1,075,000
		MM6	966,600	1,075,000
	High	MM7	117,500	235,000
		MM8	211,500	235,000
		MM9	327,500	655,000
		MM10	589,500	655,000
		MM11	537,000	1,075,000
		MM12	966,600	1,075,000
Reversion	Moderate	R1	235,000	1,095,000
		R2	655,000	1,095,000
		R3	1,075,000	1,095,000

Parameters used in coalescent simulations for models evaluated in this study. See text for migration rate parameter values; divergence times are measured in years. See figure 1 for definitions of T_1 _and T_2_.

### Model evaluation

**Figure 2:**
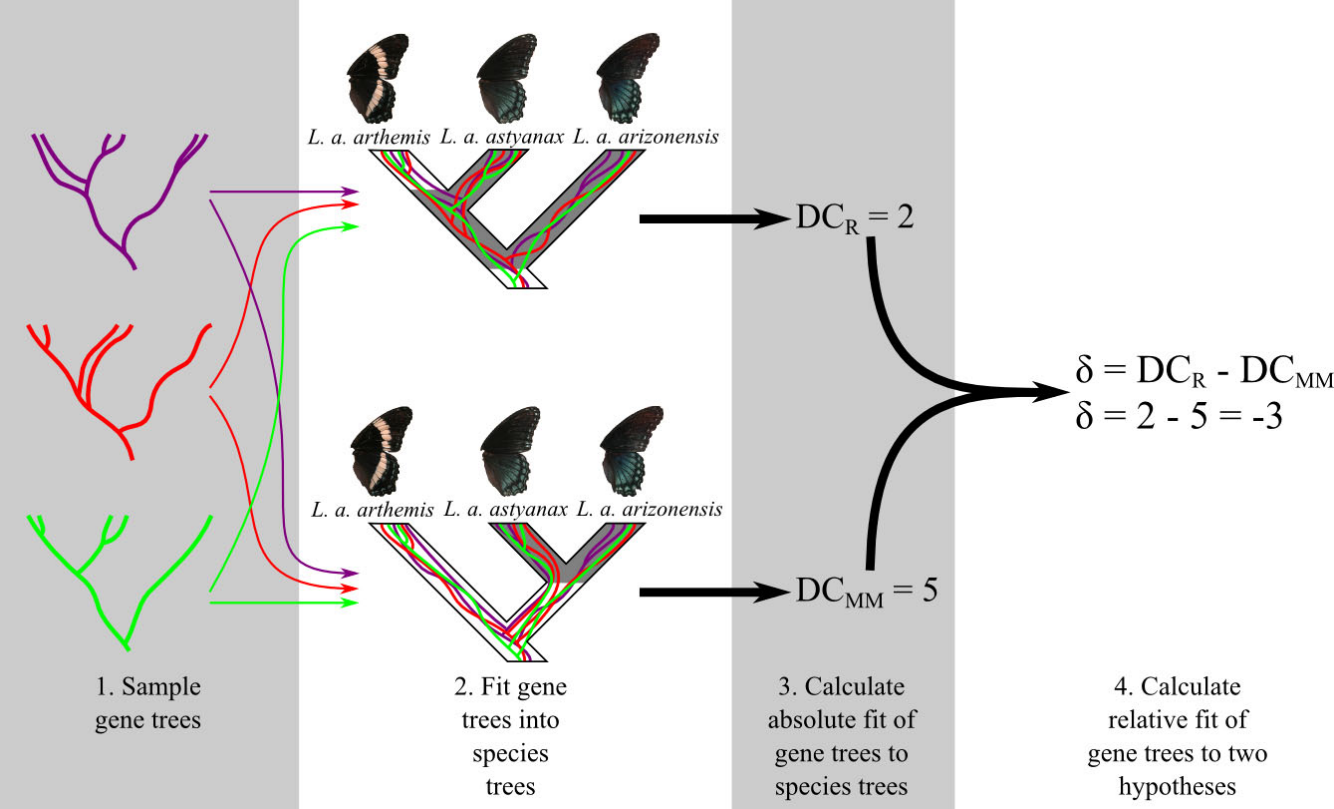
**Description of two statistics used in this study**. In the first step (1), one gene tree is sampled for each locus; for observed data, these would be estimated gene trees or trees sampled from a posterior distribution; for simulated data, trees for each locus would be simulated under identical models of population structure. These trees, one from each locus, are then fit into each of the hypotheses being tested (2). The measure of fit for the sampled gene trees is calculated for each species tree hypothesis (3); in this case, the minimum number of deep coalescences is used to measure the absolute fit of gene trees to each species trees. Finally, the difference in the two measures of absolute fit is used as the relative measure of fit to the two hypotheses being tested (4).

Each model was evaluated for two criteria, each corresponding to a particular test statistic: (1) the relative fit of the simulated data to the two hypotheses of evolution in *L. arthemis *(figure 1) and (2) the absolute fit of the simulated data to the species tree topology being tested. Support for models was determined by whether the simulated distribution of the test statistics reflected as good or better fit to hypotheses as observed in empirical data. The first test statistic, δ, measuring the relative fit of the gene trees to the two hypotheses of *L. arthemis*, was calculated as the difference in the minimum number of deep coalescences [21] between a species tree in which *L. a. arthemis *and *L. a. astyanax *are sister taxa and a species tree in which *L. a. arizonensis *and *L. a. astyanax *are sister taxa (figure 2). For the eight nuclear loci included in this study, a species tree with *L. a. astyanax *sister to *L. a. arthemis *provided a better fit than did a species tree with *L. a. astyanax *sister to *L. a. arizonensis *(93 versus 108 deep coalescences, respectively). The observed value of the test statistic δ is thus 93-108 = -15, reflecting a better fit of the *L. a. arthemis *+ *L. a. astyanax *tree (figure 1a) to the observed sequence data.

As a second metric of support for the models of *Limenitis *evolution, we calculated the minimum number of deep coalescences under a species tree corresponding to the topology of the model being tested. That is, for MM models, we calculated the minimum number of deep coalescences in a species tree with a *L. a. arizonensis *+ *L. a. astyanax*, while for R models, we calculated the minimum number of deep coalescences in a species tree with *L. a. arthemis *+ *L. a. astyanax*. It is important to note that the two test statistics (the difference in the number of deep coalescences, δ, and the absolute number of deep coalescences) are not independent, but both are necessary to measure support for each model. The former is necessary to evaluate if, under a particular model, one topological relationship is better supported than another, while second is used to determine if the simulated gene trees have been generated under models that could represent the true evolutionary history. Only models in which the observed values of both test statistics fell within the 95% simulated distributions were considered supported. Models in which the 95% simulated distribution did not include observed values were rejected. All statistics were calculated using the Mesquite software system [22].

## Results

A summary of the support for each model is shown in table 3 and Additional Files 5 and 6. None of the twelve models of a mimetic clade within *L. arthemis *corresponding to the monophyletic mimic hypothesis (MM models) were supported as none of these models produced simulated results which fulfilled both criteria for support. All MM models produced gene trees with poorer fit for a species tree with *L. a. astyanax *sister to *L. a. arthemis *than observed in empirical gene trees. That is, values for δ were consistently higher in MM models than our observed value of δ = -15, although for some MM models the 95% simulated distribution of the absolute fit (minimum number of deep coalescences) did include observed values (models MM3-MM6, MM9-MM12).

**Table 3:** Support for models of *Limenitis *history

Hypothesis	Model	δ (p)	Deep Coalescences (p)
Monophyletic Mimic	MM1	8.07 (0.0005)	156.78 (<0.0005)
	MM2	0.35 (0.015)	157.08 (<0.0005)
	MM3	12.48 (<0.0005)	**110.13 (0.438)**
	MM4	2.33 (0.001)	**108.88 (0.492)**
	MM5	13.77 (<0.0005)	**95.94 (0.9195)**
	MM6	2.96 (<0.0005)	**94.13 (0.9325)**
	MM7	5.14 (0.0015)	164.09 (<0.0005)
	MM8	-2.52 (0.032)	164.36 (<0.0005)
	MM9	7.47 (<0.0005)	**124.66 (0.0615)**
	MM10	-2.84 (0.0255)	**123.46 (0.086)**
	MM11	6.87 (0.0005)	**113.96 (0.3105)**
	MM12	-3.38 (0.025)	**112.45 (0.391)**
Reversion	R1	-29.69 (0.0025)	126.12 (<0.0005)
	R2	**-12.34 (0.316)**	**101.47 (0.173)**
	R3	-0.23 (0.0025)	**96.27 (0.381)**

Simulated values of test statistics for each of the fifteen models evaluated in this study. δ is the measure of relative fit of the model to the two hypotheses (figures 1 and 2); while Deep Coalescences measures the absolute fit of the simulated gene trees to the model tree used for simulation. Values in bold indicate observed value of statistic fell within the 95% simulated distribution. P-values represent probability that simulated gene trees fit hypotheses as well or better than observed data. See text and table 2 for details of analyses.

Of the three models corresponding to the reversion hypothesis (R models), only one, R2, was supported by both criteria. This model produced gene trees which fit a *L. a. arthemis *+ *L. a. astyanax *tree as well as observed data, and the absolute fit to this species tree matched the observed gene trees. One model, R1, with a very recent divergence of *L. a. arthemis *and *L. a. astyanax*, produced gene trees which showed a significantly better fit to a *L. a. astyanax *+ *L. a. arthemis *species tree than did the observed gene tree estimates, and thus was not supported. The divergence of the mimetic from non-mimetic lineage in model R1 was based on the divergence time estimate of Mullen et al. [16]. Conversely, an older divergence time model, R3, based on mitochondrial DNA divergences, produced gene trees which showed a worse fit to a *L. a. astyanax *+ *L. a. arthemis *tree than did the observed gene tree estimates. In models R2 and R3, with *L. a. astyanax *sister to *L. a. arthemis*, the 95% distribution of the absolute fit (the minimum number of deep coalescences) included the observed value (table 3).

## Discussion

A sister-taxa relationship between non-mimetic *L. a. arthemis *and mimetic *L. a. astyanax *was supported by our analyses. (table 3, Additional Files 5 and 6). This topology is expected under the reversion hypotheses, in which the mimetic form evolved once and was subsequently lost in *L. a. arthemis *(figure 1a) [4]. The only model to fulfill both criteria, R2, corresponds to a sister-taxa relationship between *L. a. arthemis *and *L. a. astyanax*, with a divergence between the two approximately 0.66 mya (table 2). Our analyses failed to support the hypothesis that the mimetic lineages of *L. arthemis *form a clade (figure 1b). In all monophyletic mimic models analyzed, gene trees predicted a worse relative fit to a species tree with *L. a. astyanax *sister to *L. a. arthemis *than the observed data. That is, the difference in the number of deep coalescences between the two model trees was less for simulated gene trees from any MM model than observed in empirical gene tree estimates (Additional File 5). Even in models with a high migration rate between *L. a. astyanax *and *L. a. arthemis *(MM7-MM12), which would increase the fit of the gene trees to a *L. a. astyanax *+ *L. a. arthemis *species tree, the simulated distribution of δ was still significantly higher than the observed value (table 3, Additional File 5). The available phylogenetic data and population parameter estimates [4,16] do not support the hypothesis that mimetic lineages of *L. arthemis *are sister taxa [17] and, along with previous studies [4,18], indicate that *L. a. astyanax *is likely sister to the non-mimetic *L. a. arthemis*.

There are a variety of reasons why the results of this and prior studies [4,18] conflict with the AFLP estimate in [17], including the high potential for homoplasy among AFLP markers [23]. Additionally, the lack of applicable evolutionary models to AFLP markers and dependence on distance-based estimates of phylogeny may result in inconsistent estimates of phylogeny, especially when terminal branches are connected by relatively short internodes [23-25]. Finally, phylogenetic estimates with low taxon sampling may be prone to inconsistency, especially when markers used for estimation are evolving relatively rapidly [26]. Alternatively, published estimates of model parameters [16] used in simulations may not accurately reflect the true history of this group, and thus the models we evaluated did not encompass sufficient parameter space. More complex parametric models, tests accommodating uncertainty in gene tree estimates [27], and increased precision in population parameters would all benefit our understanding of how mimetic phenotypes arise and change over time.

Did the white-banded *L. a. arthemis *evolve from a mimetic lineage, as proposed in the reversion hypothesis? In light of the phylogenetic tests on multi-locus data presented here, this remains a plausible explanation for the observed data. The mimetic phenotype is hypothesized to have arisen in the lineage which eventually gave rise to all *L. arthemis *taxa; in areas where the model, *B. philenor *was absent, the mimetic phenotype was lost, giving rise to the disruptively colored *L. a. arthemis*. Selection against mimetic phenotypes in the absence of the model is predicted to favor phenotypes with alternative defensive strategies, such as disruptive coloration, and may occur in other systems, such as king snakes [23]. Although it remains possible that the monophyletic mimic hypothesis [17] is correct, or that mimicry evolved twice in the *L. arthemis *lineage [18], additional data would be needed to support these hypotheses.

The key to understanding the evolution of mimicry within this group will require integration of geographical and developmental approaches. Extensive geographical sampling and geographically explicit models of population structure will be necessary to determine the extent of gene flow between *L. a. arthemis *and *L. a. astyanax*, and the degree to which introgression causes discordance between gene trees and species trees. The biogeographical history of all *L. arthemis *lineages, based on increased sampling of *L. a. arizonensis*, along with detailed history of the model, *B. philenor*, will be necessary for a better understanding of mimicry evolution [4]. Future work should also couple the phylogenetic estimates of ancestry with a developmental genetic approach assessing homology among the various mimetic and non-mimetic phenotypes. The identity and history of the genetic loci responsible for the respective phenotypes will prove invaluable in studying the evolution of mimicry [18,28]. This approach will be necessary to distinguish among the various hypotheses regarding the history underlying the non-mimetic phenotype of *L. a. arthemis*, including, but not limited to: a loss of function mutation in the mimetic coloration network resulting in a reversion to an ancestral phenotype; a gain of function mutation representing novel evolution of the white-banded phenotype; or a gain of disruptive coloration via adaptive introgression with other North American white-banded lineages (*L. weidemeyerii*, *L. lorquini*).

## Conclusions

Hypothesis tests based on mitochondrial [4] and nuclear loci (this study), which explicitly evaluated support for a mimetic clade within *L. arthemis*, rejected the hypothesis that *L. a. astyanax *and *L. a. arizonensis *are sister taxa [17] and found support only for a phylogeny consistent with the reversion hypothesis (figure 1a). These results also demonstrate how studying character evolution requires an understanding of the basis of and limitations to phylogenetic tree estimation. The future of the comparative approach lies in accommodating deterministic and stochastic processes responsible for the observed patterns of biological diversity. The possibility of different processes giving rise to identical patterns must be accounted for, and the relative support for those processes can be measured in a quantitative framework as presented here. This study presents a modest attempt at evaluating the different evolutionary models of the mimetic phenotype, and the phylogenetic analyses presented here provide a framework for future investigations of the evolution of mimicry. With the current computational resources available, studies should move away from relying solely on phylogenetic point estimates (e.g., single trees) for comparing processes of character evolution. Instead, we should use model-based approaches, such as parametric bootstrapping, to assess the relative support of alternative evolutionary hypotheses. By comparing the variation underlying the observed pattern of diversity, we can gain deeper insights into the likelihood of various evolutionary processes.

## Authors' contributions

JCO and KLP designed the study. JCO developed and performed analyses. JCO and KLP drafted the manuscript. All authors read and approved the final manuscript.

## Supplementary Material

Additional file 1 **GenBank accession numbers for sequences used in this study**. GenBank accession numbers for all genetic data used in phylogenetic tree estimation. Click here for file

Additional file 2 **Gene tree estimation and parameter estimates**. Details of Bayesian gene tree estimation and sources of parameters used in simulations. Click here for file

Additional file 3 **Gene tree estimates of eight nuclear loci**. Bayesian phylogenies of North American *Limenitis *taxa. Click here for file

Additional file 4 **Models of North American *Limenitis *divergences**. Schematic of relationships among North American *Limenitis *lineages used in coalescent simulations. Click here for file

Additional file 5 **Simulated distributions of the test statistic δ**. Frequency distribution for the test statistic δ simulated in 15 models of population structure. Click here for file

Additional file 6 **Simulated distributions of the minimum number of deep coalescences**. Frequency distribution for the number of deep coalescences simulated in 15 models of population structure. Click here for file
