# Supplementary material for: Are mimics monophyletic? The necessity of phylogenetic hypothesis tests in character evolution
Source: BMC Evol Biol. 2010 Aug 3;10:239. doi: 10.1186/1471-2148-10-239 (PMC3020633; doi:10.1186/1471-2148-10-239)
Supplement: Additional file 1 — GenBank accession numbers for sequences used in this study. GenBank accession numbers for all genetic data used in phylogenetic tree estimation. [file 1471-2148-10-239-S1.PDF]

Additional File 1 for Oliver & Prudic, “Are mimics monophyletic? The necessity of phylogenetic hypothesis tests in character evolution.”

GenBank accession numbers for sequences used in this study.

Taxon	Anon6	Anon10	Anon15	Anon17
L. archippus	EU125180	EU125130	EU125081	-
L. arthemis arizonensis	EU125161, EU125162	EU125111, EU125112	EU125061	EU125135, EU125136
L. arthemis arthemis	EU125168, EU125169, EU125171, EU125172, EU125173, EU125159, EU125160, EU125164, EU125174, EU125175, EU125176	EU125117, EU125119, EU125120, EU125121, EU125122, EU125123, EU125110, EU125114, EU125124, EU125125, EU125126	EU125060, EU125063, EU125066, EU125067, EU125069, EU125070, EU125071, EU125072, EU125073, EU125075, EU125076	EU125142, EU125143, EU125145, EU125146, EU125147, EU125148, EU125149, EU125134, EU125138, EU125150, EU125151, EU125152, EU125153
L. arthemis astyanax	EU125163, EU125165, EU125166, EU125170, EU125177, EU125178, EU125179	EU125113, EU125115, EU125118, EU125127, EU125128, EU125129, EU125131, EU125132	EU125062, EU125064, EU125065, EU125068, EU125074, EU125077, EU125078, EU125079, EU125080	EU125137, EU125139, EU125140, EU125144, EU125154, EU125155, EU125156, EU125157
L. lorquini	EU125167	EU125116	-	EU125141
L. weidemeyerii	EU125181	EU125133	EU125082	EU125158

Taxon	EF1α	Kettin
L. archippus	DQ208217, EF643262, EF643263, EF643264, EF643265, EF643266, EF643267, EF643268, EF643269, EF643270, EF643271, EF643272, EF643273, EF643274, EF643275, EF643276, EF643277, EF643278, EF643279, EF643280, EF643281, EF643282	EU125092
L. arthemis arizonensis	DQ208215, EF643283, EF643284, EF643285, EF643286, EF643287, EF643288, EF643289, EF643290, EF643291, EF643292, EF643293	EU125084, EU125085, EU125086, EU125087
L. arthemis arthemis	DQ208218, EF643294, EF643295, EF643296, EF643297, EF643298, EF643299, EF643300, EF643316, EF643317, EF643318, EF643319, EF643320, EF643321	EU125095, EU125096, EU125098, EU125099, EU125100, EU125101, EU125083, EU125089, EU125102, EU125103, EU125104
L. arthemis astyanax	DQ208219, EF643301, EF643302, EF643303, EF643304, EF643305, EF643306, EF643307, EF643308, EF643309, EF643310, EF643311, EF643312, EF643313, EF643314, EF643315	EU125088, EU125090, EU125091, EU125097, EU125105, EU125106, EU125107, EU125108, EU125109
L. lorquini	DQ208225, EF643326, EF643327, EF643328, EF643329, EF643330, EF643331, EF643332, EF643333, EF643334, EF643335, EF643336, EF643337, EF643338, EF643339, EF643340, EF643341	EU125093
L. weidemeyerii	DQ208229, EF643347, EF643348, EF643349, EF643350, EF643351, EF643352, EF643353, EF643354, EF643355, EF643356, EF643357, EF643358, EF643359, EF643360	EU125094

Taxon	Ldh	wg
L. archippus	EU125056	EU433935, EU433936, EU433937, EU433938, EU098279, EU098278, EU098277
L. arthemis arizonensis	EU125035, EU125036, EU125037	EU433939, EU098263
L. arthemis arthemis	EU125034, EU125039, EU125043, EU125044, EU125046, EU125047, EU125048, EU125049, EU125050, EU125051, EU125052	EU433940, EU098275, EU098274, EU098273, EU433942, EU098250, EU098249, EU098248, EU098258, EU098257, EU098256, EU098255, EU098254, EU098253, EU098252, EU098251
L. arthemis astyanax	EU125038, EU125040, EU125041, EU125045, EU125053, EU125054, EU125055, EU125057, EU125058	EU433941, EU098271, EU098270, EU098266
L. lorquini	EU125042	EU433944
L. weidemeyerii	EU125059	EU433946, EU098272, EU098269, EU098264
