# Supplementary material for: Are mimics monophyletic? The necessity of phylogenetic hypothesis tests in character evolution
Source: BMC Evol Biol. 2010 Aug 3;10:239. doi: 10.1186/1471-2148-10-239 (PMC3020633; doi:10.1186/1471-2148-10-239)
Supplement: Additional file 2 — Gene tree estimation and parameter estimates. Details of Bayesian gene tree estimation and sources of parameters used in simulations. [file 1471-2148-10-239-S2.PDF]

Additional File 2 for Oliver & Prudic, "Are mimics monophyletic? The necessity of phylogenetic hypothesis tests in character evolution."

Gene tree estimates

For the eight loci, we used GenBank sequences (additional file 1) in Bayesian MCMC searches [1]. For each locus, we performed likelihood ratio tests [2] to select the best-fit model of evolution for Bayesian estimation. Models used in Bayesian tree estimation were as follows: HKY+Γ: *Anon6*, *Anon15*; HKY+I: *EF1α*; GTR+Γ: *Anon10*, *Anon17*, *kettin*, *Ldh*, *wg*. For all loci we performed two MCMC runs of four chains each, sampling every 1000 generations; trees sampled before the two runs had converged were discarded. Runs were considered converged when the standard deviation of the split frequencies dropped below 0.02 [1]. In seven analyses (all loci except *Anon17*), convergence had occurred by the 1 millionth generation, so 2 million generations were run and trees sampled in the second half of the MCMC search were used to generate a consensus tree for subsequent analyses. For the *Anon17* data, the two MCMC runs did not converge until the 7 millionth generation; we continued the run for a total of 8 million generations, sampling trees from only the final million generations. Consensus trees for all loci are shown in additional file 3. All gene tree estimates were congruent with previous studies [3-5].

Divergence time estimates

For all models tested, divergence of the *L. arthemis* lineages from the three other North American lineages (*L. archippus*, *L. lorquini*, *L. weidemeyerii*), as well as divergences among those three lineages, were based on average pairwise divergences of mitochondrial cytochrome oxidase subunits I and II (COI and COII, respectively). Assuming a rate of 2.3% sequence divergence per million years [6], divergence estimates are given in additional file 4. For divergences among lineages of *L. arthemis*, we used three different estimates for the divergence of *L. a. arthemis* from its sister taxon (*L. a. astyanax* in 'R' models, T_1 in Figure 1*a*; a clade of *L. a. astyanax* and *L. a. arizonensis* in 'MM' models, T_2 in Figure 1*b*). The most recent estimate is 235,000 ybp, based on the estimate from [5]. The deepest estimate is 1,075,000 ybp, based on pairwise sequence divergence estimates of mitochondrial genes COI and COII. Finally, an estimate of 655,000 ybp is based on the arithmetic mean of the two previous estimates. For 'MM' models, in which *L. a. astyanax* and *L. a. arizonensis* are sister taxa, we used divergence times between those two taxa that were 0.5 and 0.9 times the divergence of *L. a. arthemis* from the ancestor of (*L. a. astyanax* + *L. a. arizonensis*), or simply $0.5T_2$ and $0.9T_2$, respectively. For 'R' models, all estimates of the divergence of *L. a. arizonensis* from the *L. a. arthemis* + *L. a. astyanax* clade are based on average pairwise sequence divergence in COI and COII of 2.513% ≈ 1,095,000 ybp.

References

1. Huelsenbeck JP, Ronquist F: **MRBAYES: Bayesian inference of phylogeny.** *Bioinformatics* 2001, **17:**754-755.

2. Sullivan J, Swofford DL: **Are guinea pigs rodents? The importance of adequate models in molecular phylogenetics.** *J Mam Evol* 1997, **4:**77-86.

3. Mullen SP: **Wing pattern evolution and the origins of mimicry among North American admiral butterflies (Nymphalidae: *Limenitis*).** *Mol Phylogenet Evol* 2006, **39:**747-758.

4. Prudic KL, Oliver JC: **Once a Batesian mimic, not always a Batesian mimic: mimic reverts back to ancestral phenotype when model is absent.** *Proc R Soc B* 2008*,* **275:**1125-1132.

5. Mullen SP, Dopman EB, Harrison RG: **Hybrid zone origins, species boundaries, and the evolution of wing-pattern diversity in a polytypic species complex of North American admiral butterflies (Nymphalidae: *Limenitis*).** *Evolution* 2008, **62:**1400-1418.

6. Brower AVZ: **Rapid morphological radiation and convergence among races of the butterfly *Heliconius erato* inferred from patterns of mitochondrial DNA evolution.** *Proc Natl Acad Sci USA* 1994, **91:**6491-6495.
