# Supplementary material for: Are mimics monophyletic? The necessity of phylogenetic hypothesis tests in character evolution
Source: BMC Evol Biol. 2010 Aug 3;10:239. doi: 10.1186/1471-2148-10-239 (PMC3020633; doi:10.1186/1471-2148-10-239)
Supplement: Additional file 3 — Gene tree estimates of eight nuclear loci. Bayesian phylogenies of North American Limenitis taxa. [file 1471-2148-10-239-S3.PDF]

Additional File 3 for Oliver & Prudic, "Are mimics monophyletic? The necessity of phylogenetic hypothesis tests in character evolution."

Gene tree estimates of eight nuclear loci: (a) *Anon6*, (b) *Anon10*, (c) *Anon15*, (d) *Anon17*, (e) *EF1α*, (f) *kettin*, (g) *Ldh*, and (h) *wg*. In all trees, branch labels are Bayesian posterior probabilities and branch lengths are measured in expected number of substitutions. Taxon icons are: (■) *L. archippus*, (●) *L. arthemis arizonensis*, (❍) *L. a. arthemis*, (●) *L. a. astyanax*, (▼) *L. lorquini*, (▲) *L. weidemeyerii.*

(a) *Anon6*

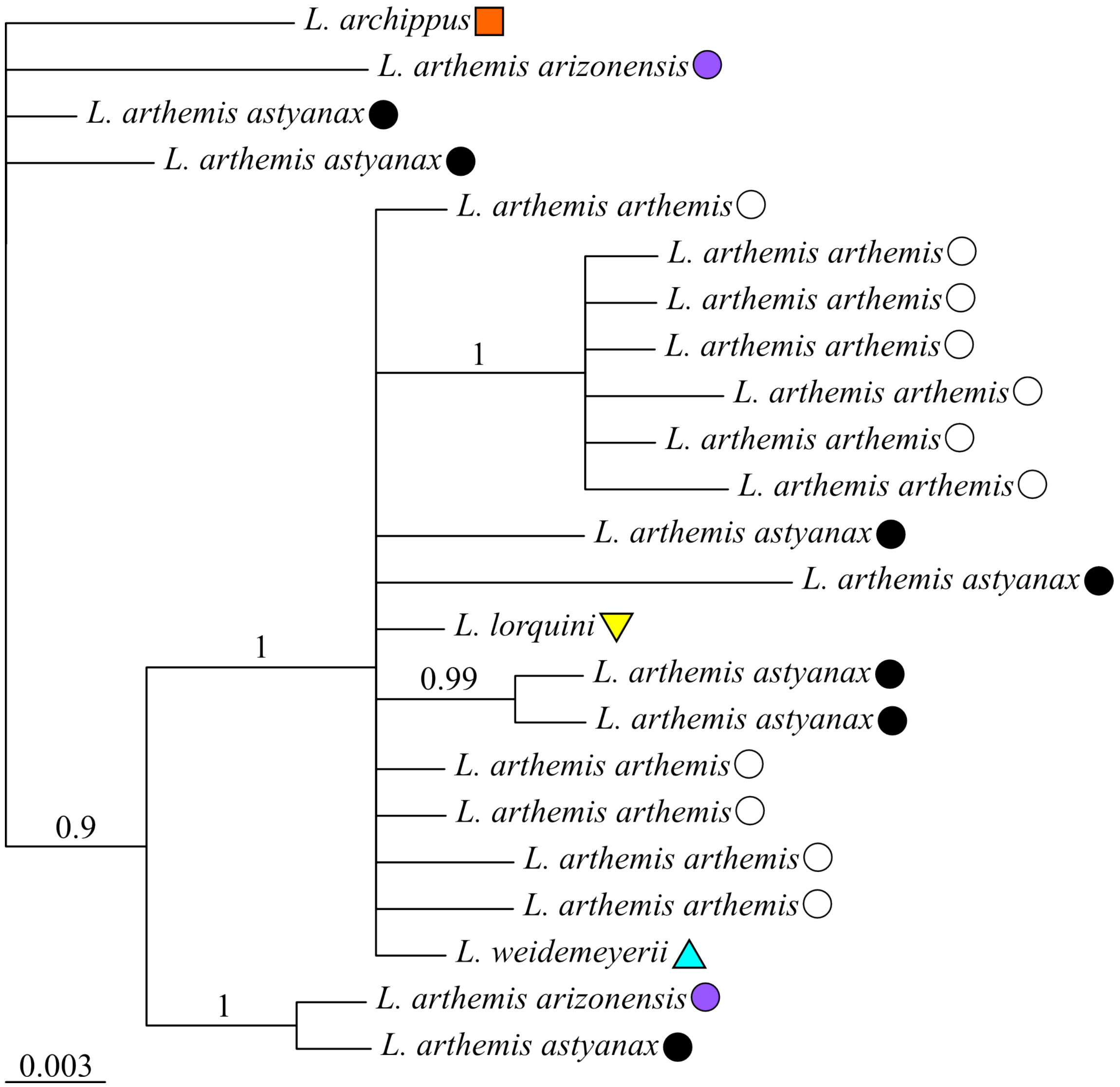

(b) *Anon10*

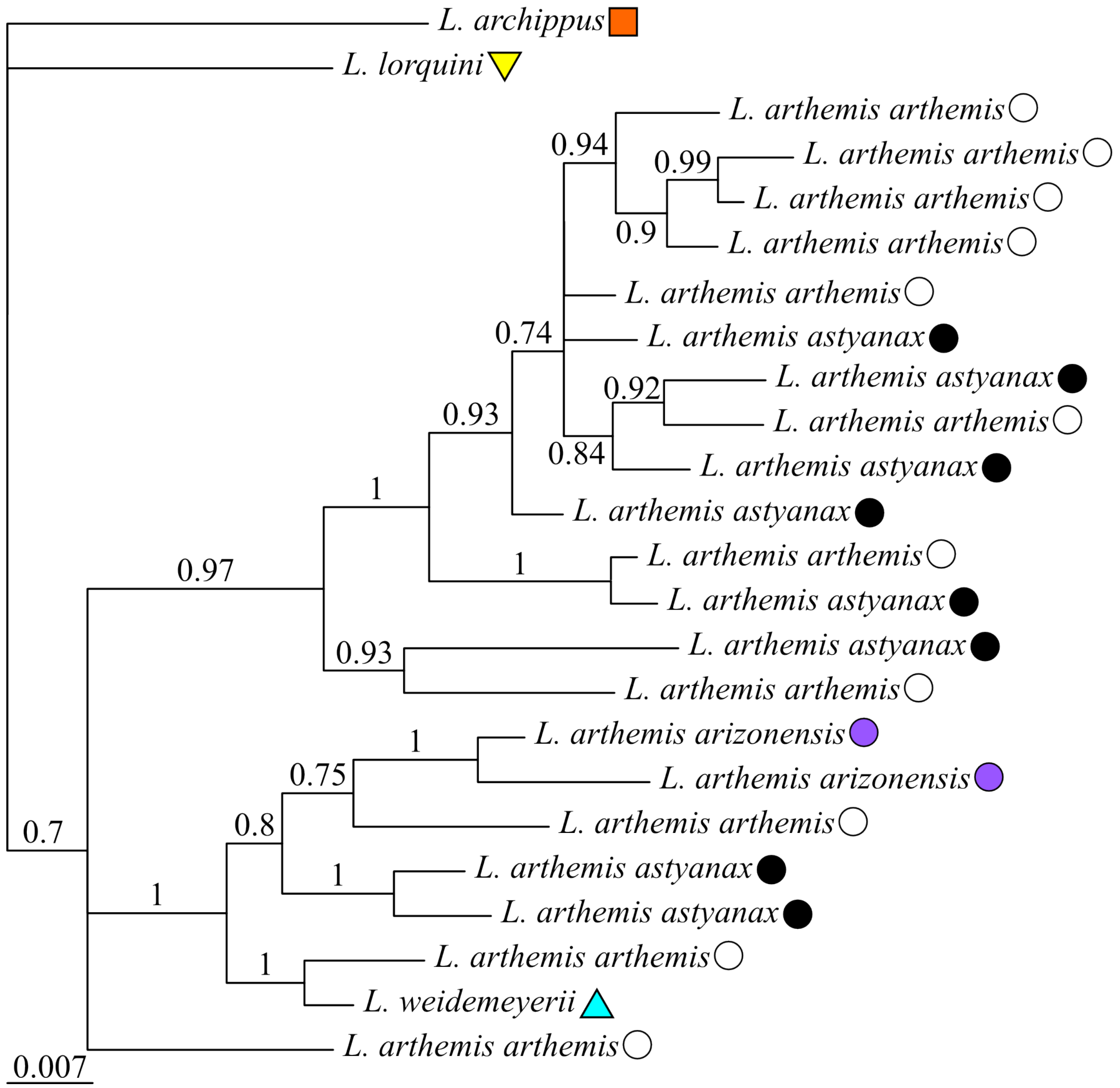
L. archippus
L. lorquini
L. arthemis arthemis
0.94
0.99
L. arthemis arthemis
L. arthemis arthemis
0.9
L. arthemis arthemis
L. arthemis arthemis
0.74
L. arthemis astyanax
0.92
L. arthemis astyanax
0.93
L. arthemis arthemis
0.84
L. arthemis astyanax
1
L. arthemis astyanax
1
L. arthemis arthemis
0.97
L. arthemis astyanax
0.93
L. arthemis astyanax
L. arthemis arthemis
1
L. arthemis arizonensis
0.75
L. arthemis arizonensis
L. arthemis arthemis
0.8
0.7
1
L. arthemis astyanax
1
L. arthemis astyanax
L. arthemis arthemis
1
L. weidemeyerii
L. arthemis arthemis
0.007

(c) *Anon15*

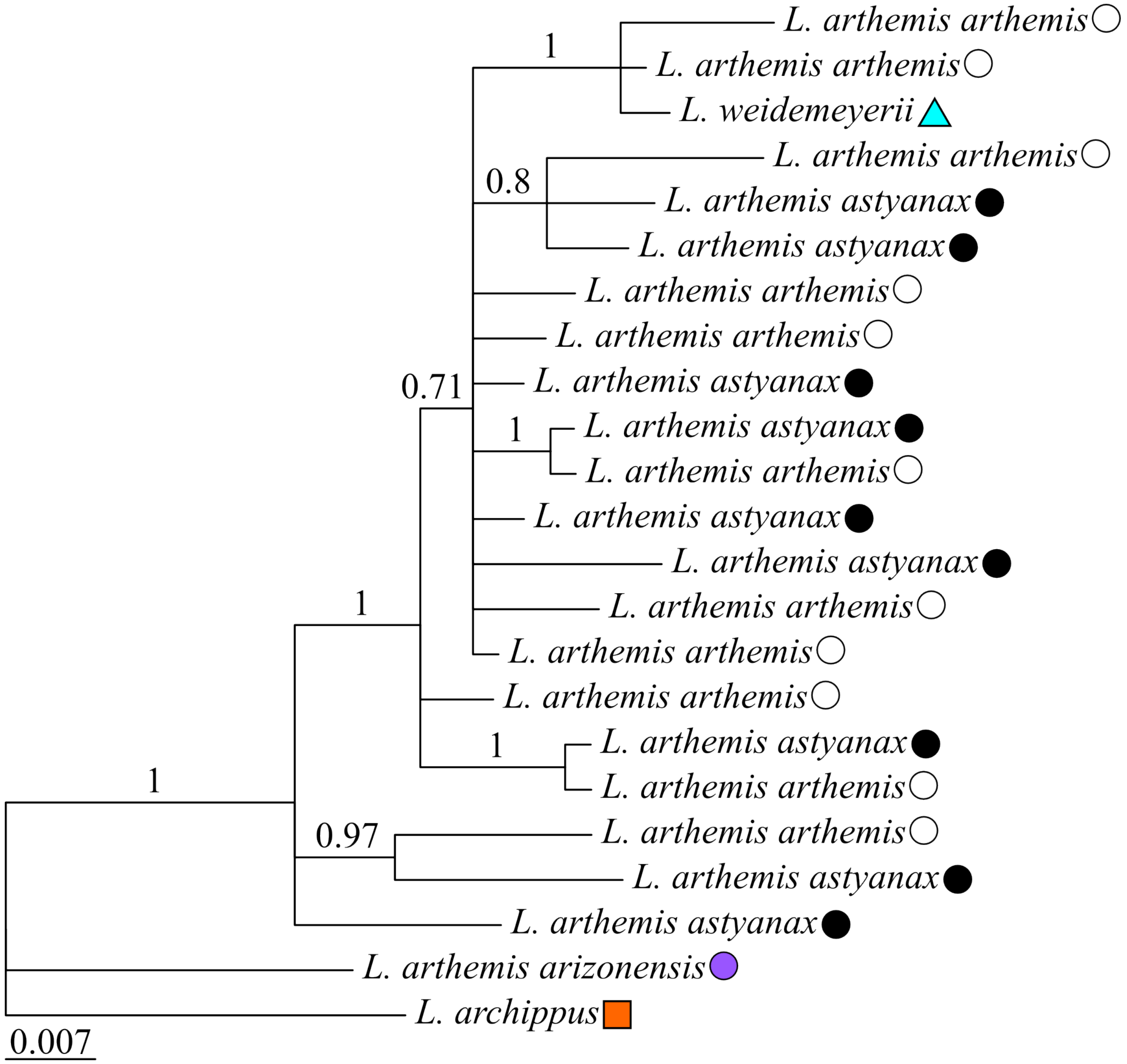

1
L. arthemis arthemis
L. arthemis arthemis
L. weidemeyerii
0.8
L. arthemis arthemis
L. arthemis astyanax
L. arthemis astyanax
L. arthemis arthemis
L. arthemis arthemis
0.71
L. arthemis astyanax
1
L. arthemis astyanax
L. arthemis arthemis
L. arthemis astyanax
L. arthemis astyanax
L. arthemis arthemis
1
L. arthemis arthemis
L. arthemis arthemis
1
L. arthemis astyanax
L. arthemis arthemis
1
0.97
L. arthemis arthemis
L. arthemis astyanax
L. arthemis astyanax
L. arthemis arizonensis
L. archippus
0.007

(d) *Anon17*

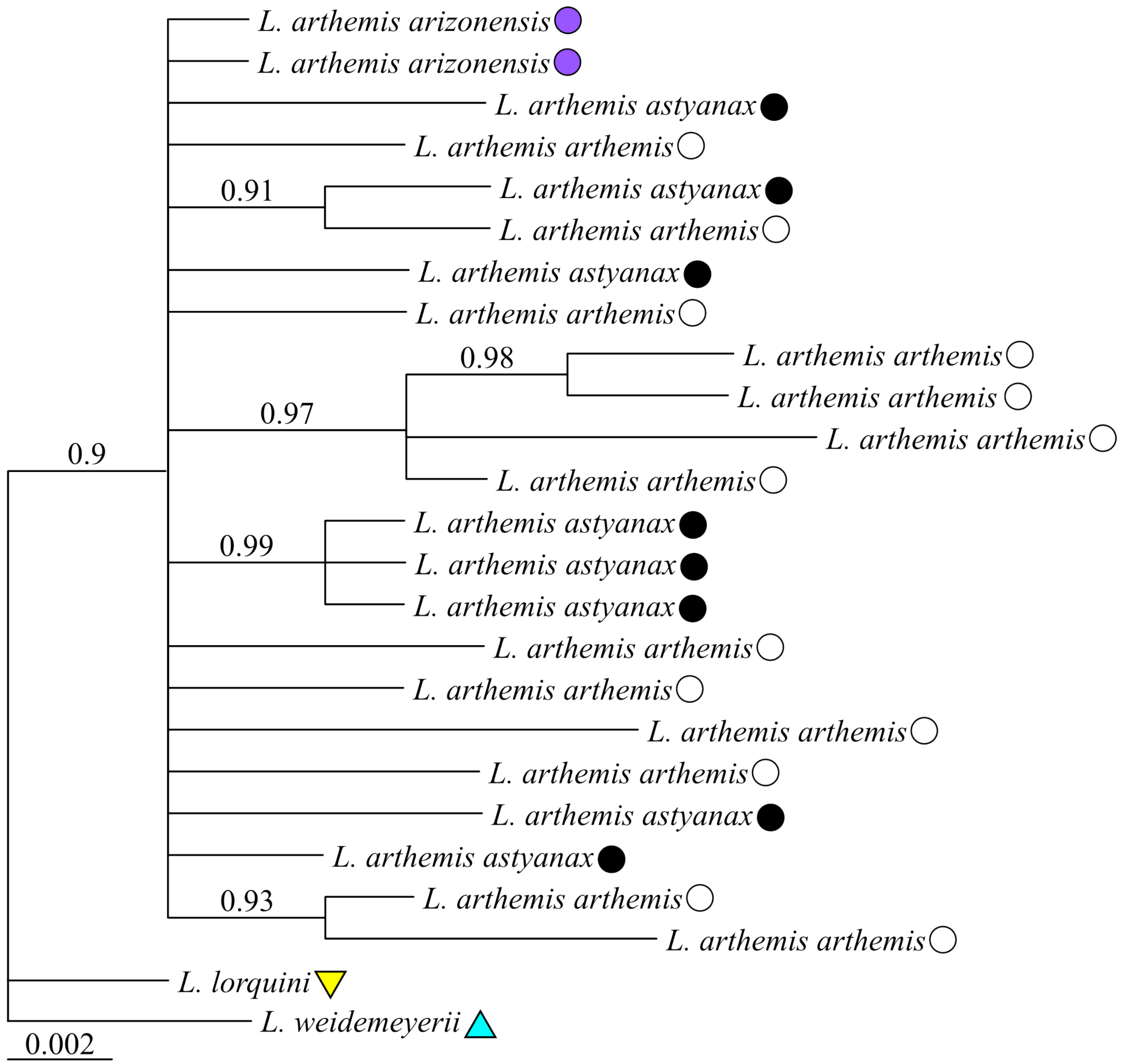

L. arthemis arizonensis
L. arthemis arizonensis
L. arthemis astyanax
L. arthemis arthemis
0.91
L. arthemis astyanax
L. arthemis arthemis
L. arthemis astyanax
L. arthemis arthemis
0.98
L. arthemis arthemis
L. arthemis arthemis
0.97
L. arthemis arthemis
0.9
L. arthemis arthemis
L. arthemis astyanax
0.99
L. arthemis astyanax
L. arthemis astyanax
L. arthemis arthemis
L. arthemis arthemis
L. arthemis arthemis
L. arthemis arthemis
L. arthemis astyanax
L. arthemis astyanax
0.93
L. arthemis arthemis
L. arthemis arthemis
L. lorquini
L. weidemeyerii
0.002

(e) *EF1α*

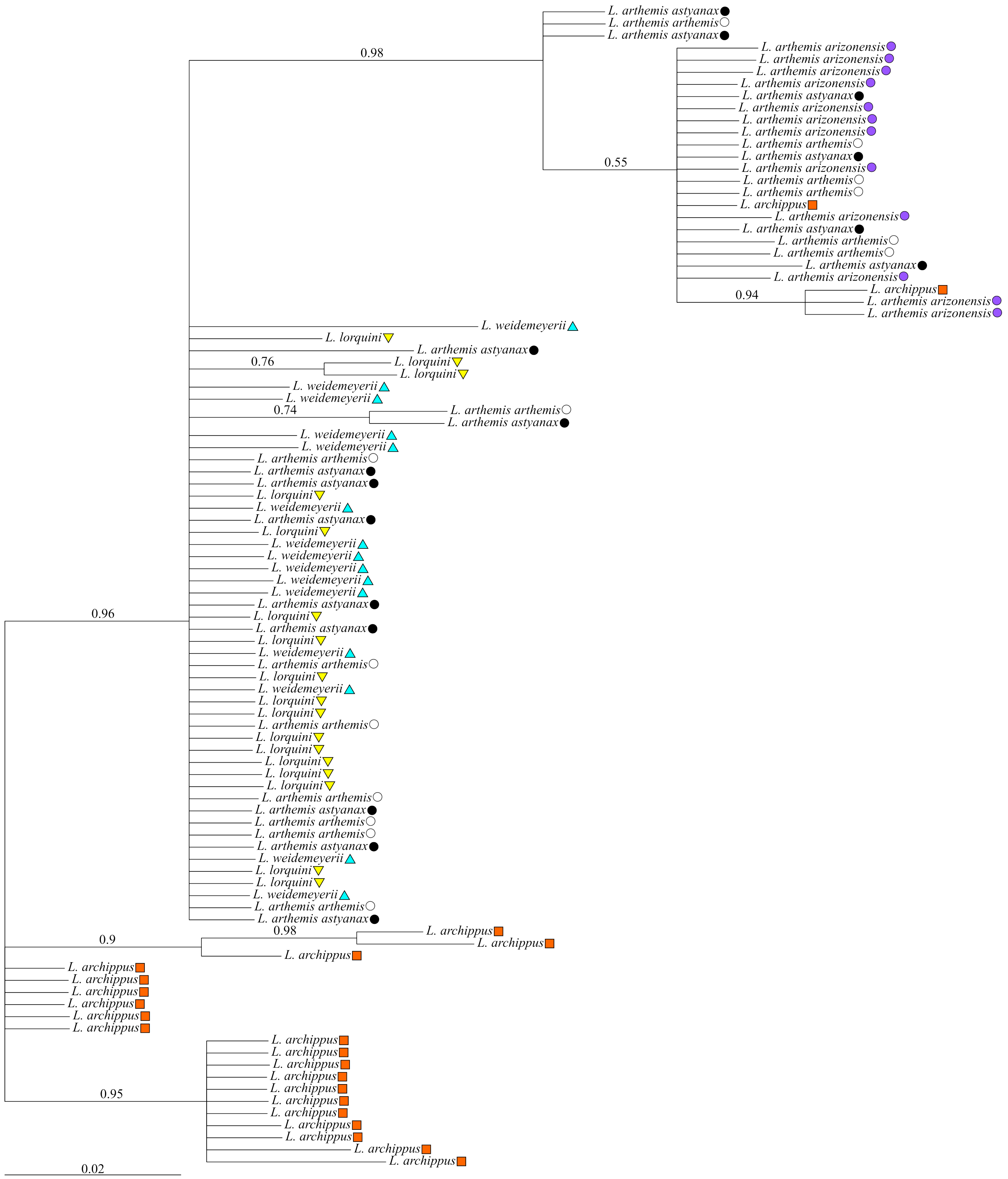

L. arthemis astyanax
L. arthemis arthemis
L. arthemis arizonensis
L. archippus
L. weidemeyerii
L. lorquini
0.98
0.55
0.94
0.76
0.74
0.96
0.9
0.95
0.02

(f) *kettin*

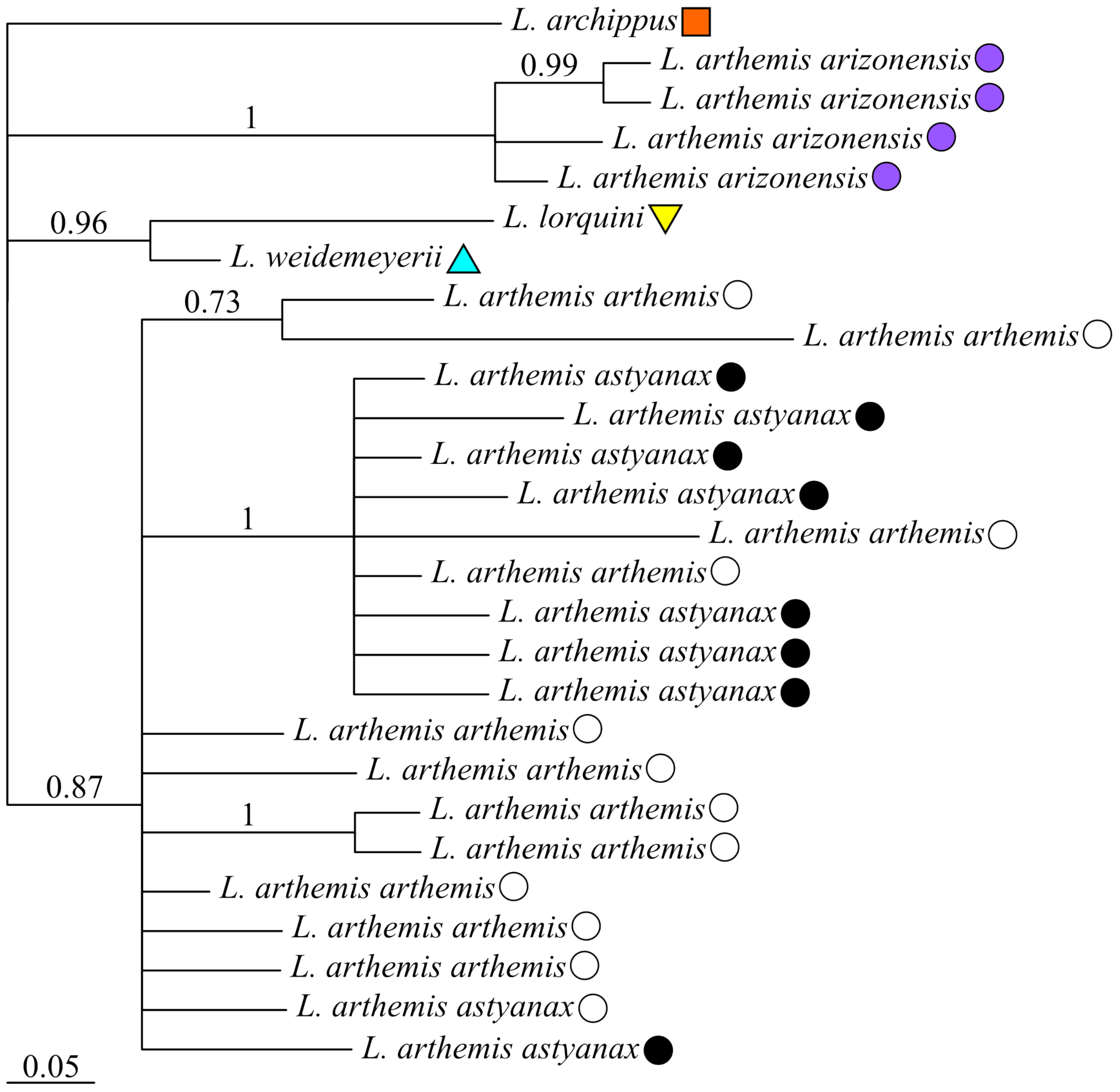

L. archippus
0.99
L. arthemis arizonensis
L. arthemis arizonensis
1
L. arthemis arizonensis
L. arthemis arizonensis
0.96
L. lorquini
L. weidemeyerii
0.73
L. arthemis arthemis
L. arthemis arthemis
L. arthemis astyanax
L. arthemis astyanax
L. arthemis astyanax
L. arthemis astyanax
1
L. arthemis arthemis
L. arthemis arthemis
L. arthemis astyanax
L. arthemis astyanax
L. arthemis astyanax
L. arthemis arthemis
L. arthemis arthemis
0.87
1
L. arthemis arthemis
L. arthemis arthemis
L. arthemis arthemis
L. arthemis arthemis
L. arthemis arthemis
L. arthemis astyanax
L. arthemis astyanax
0.05

(g) *Ldh*

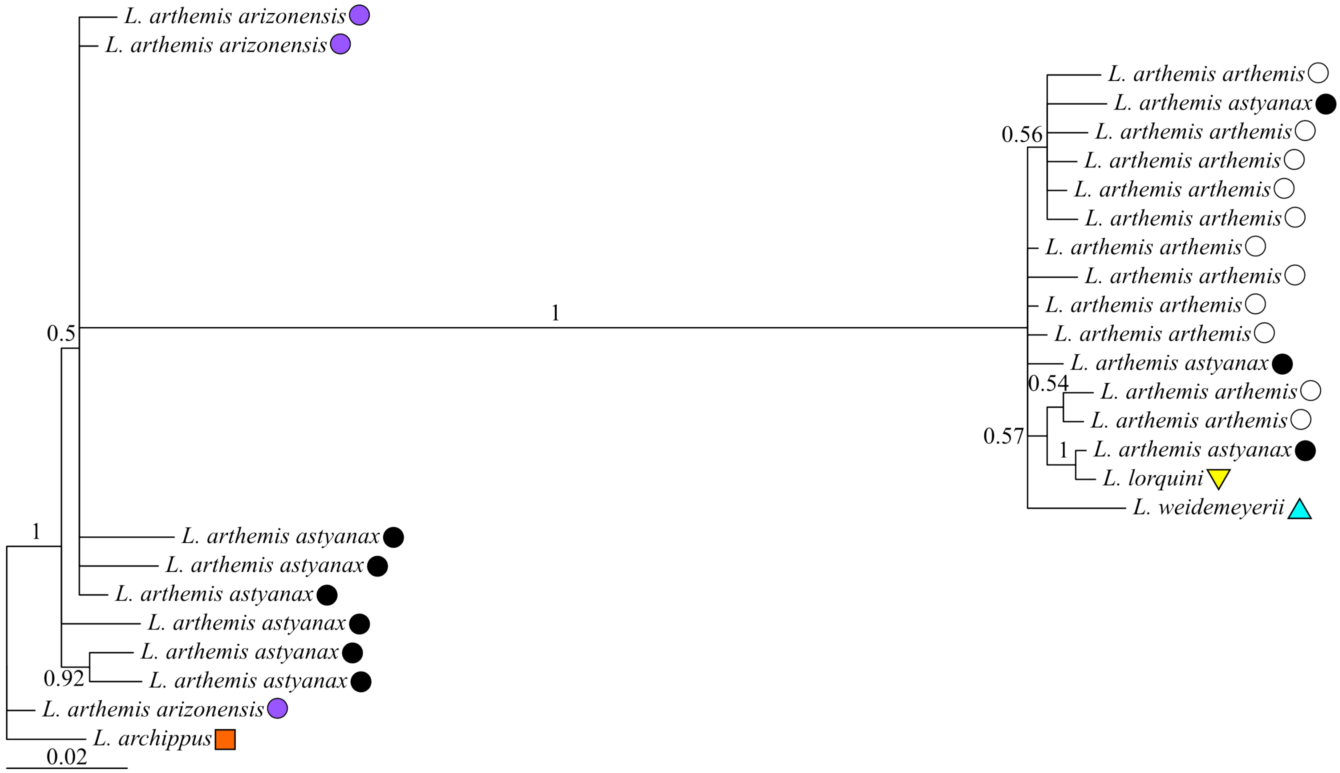

L. arthemis arizonensis
L. arthemis arizonensis
L. arthemis arthemis
L. arthemis astyanax
L. arthemis arthemis
L. arthemis arthemis
L. arthemis arthemis
L. arthemis arthemis
0.56
L. arthemis arthemis
L. arthemis arthemis
L. arthemis arthemis
L. arthemis arthemis
1
0.5
L. arthemis astyanax
0.54
L. arthemis arthemis
L. arthemis arthemis
0.57
1
L. arthemis astyanax
L. lorquini
L. weidemeyerii
1
L. arthemis astyanax
L. arthemis astyanax
L. arthemis astyanax
L. arthemis astyanax
L. arthemis astyanax
0.92
L. arthemis astyanax
L. arthemis arizonensis
L. archippus
0.02

(h) *wg*

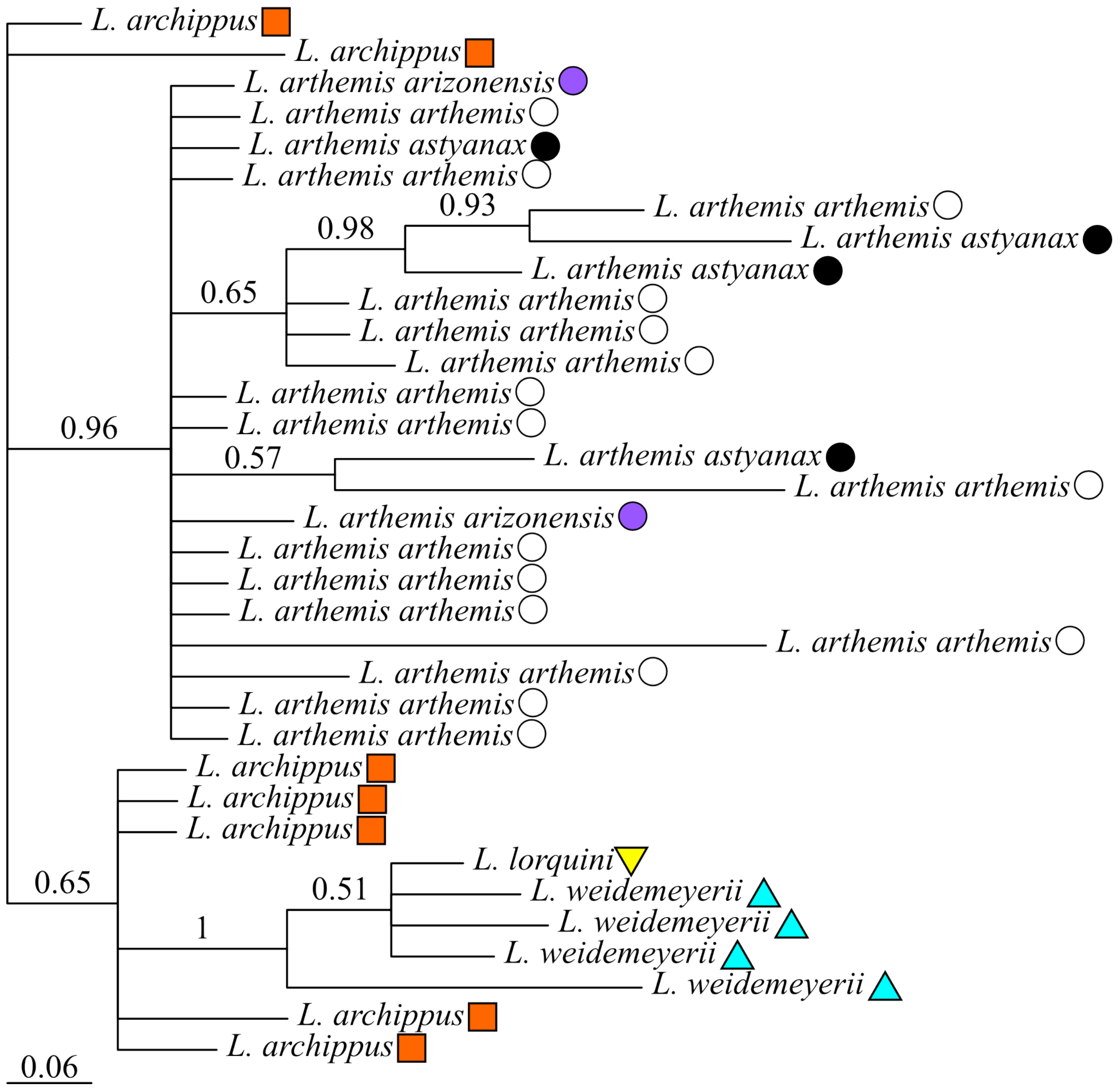
L. archippus
L. archippus
L. arthemis arizonensis
L. arthemis arthemis
L. arthemis astyanax
L. arthemis arthemis
0.93
L. arthemis arthemis
L. arthemis astyanax
0.98
L. arthemis astyanax
0.65
L. arthemis arthemis
L. arthemis arthemis
L. arthemis arthemis
L. arthemis arthemis
0.96
L. arthemis arthemis
0.57
L. arthemis astyanax
L. arthemis arthemis
L. arthemis arizonensis
L. arthemis arthemis
L. arthemis arthemis
L. arthemis arthemis
L. arthemis arthemis
L. arthemis arthemis
L. arthemis arthemis
L. arthemis arthemis
L. archippus
L. archippus
L. archippus
L. lorquini
0.51
L. weidemeyerii
0.65
L. weidemeyerii
1
L. weidemeyerii
L. weidemeyerii
L. archippus
L. archippus
0.06
