# Supplementary material for: Are mimics monophyletic? The necessity of phylogenetic hypothesis tests in character evolution
Source: BMC Evol Biol. 2010 Aug 3;10:239. doi: 10.1186/1471-2148-10-239 (PMC3020633; doi:10.1186/1471-2148-10-239)

Additional File 4 for Oliver & Prudic, "Are mimics monophyletic? The necessity of phylogenetic hypothesis tests in character evolution."

Schematic of relationships among North American *Limenitis* lineages for coalescent simulations. Divergence times used in simulations are as follows: split between *L. lorquini* and *L. weidemeyerii*, T_3 = 945,000 ybp; *L. arthemis* divergence from *L. lorquini* + *L. weidemeyerii*, T_4 = 1,275,000 ybp, divergence of *L. archippus* from remaining North American *Limenitis*, T_5 = 1,870,000 ybp. See table 2 and figure 1 for divergences among *L. arthemis* lineages.

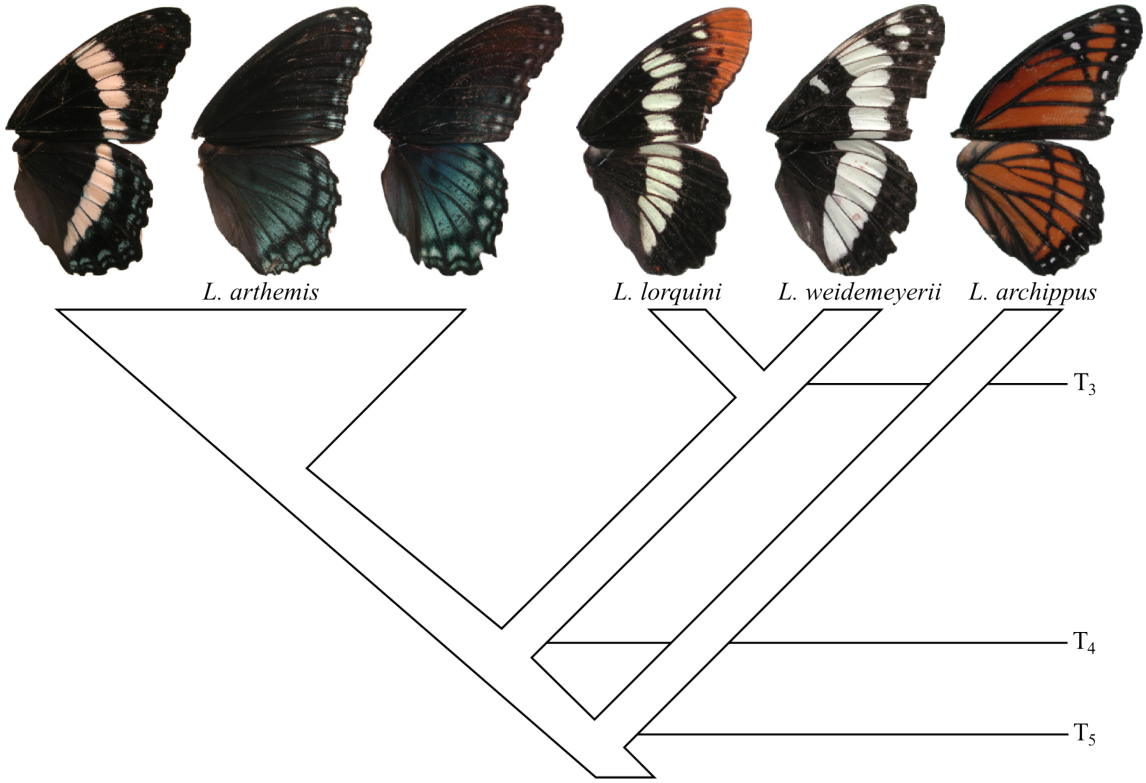

Supplement: Additional file 4 — Models of North American Limenitis divergences. Schematic of relationships among North American Limenitis lineages used in coalescent simulations. [file 1471-2148-10-239-S4.PDF]
