# Supplementary material for: Are mimics monophyletic? The necessity of phylogenetic hypothesis tests in character evolution
Source: BMC Evol Biol. 2010 Aug 3;10:239. doi: 10.1186/1471-2148-10-239 (PMC3020633; doi:10.1186/1471-2148-10-239)

Additional File 5 for Oliver & Prudic, "Are mimics monophyletic? The necessity of phylogenetic hypothesis tests in character evolution."

Simulated distributions of the test statistic δ. Arrows indicate observed value; shaded area represents the upper 95% distribution in unsupported models (except model R1, where shaded area represents the lower 95% distribution). Model details are found in table 2.

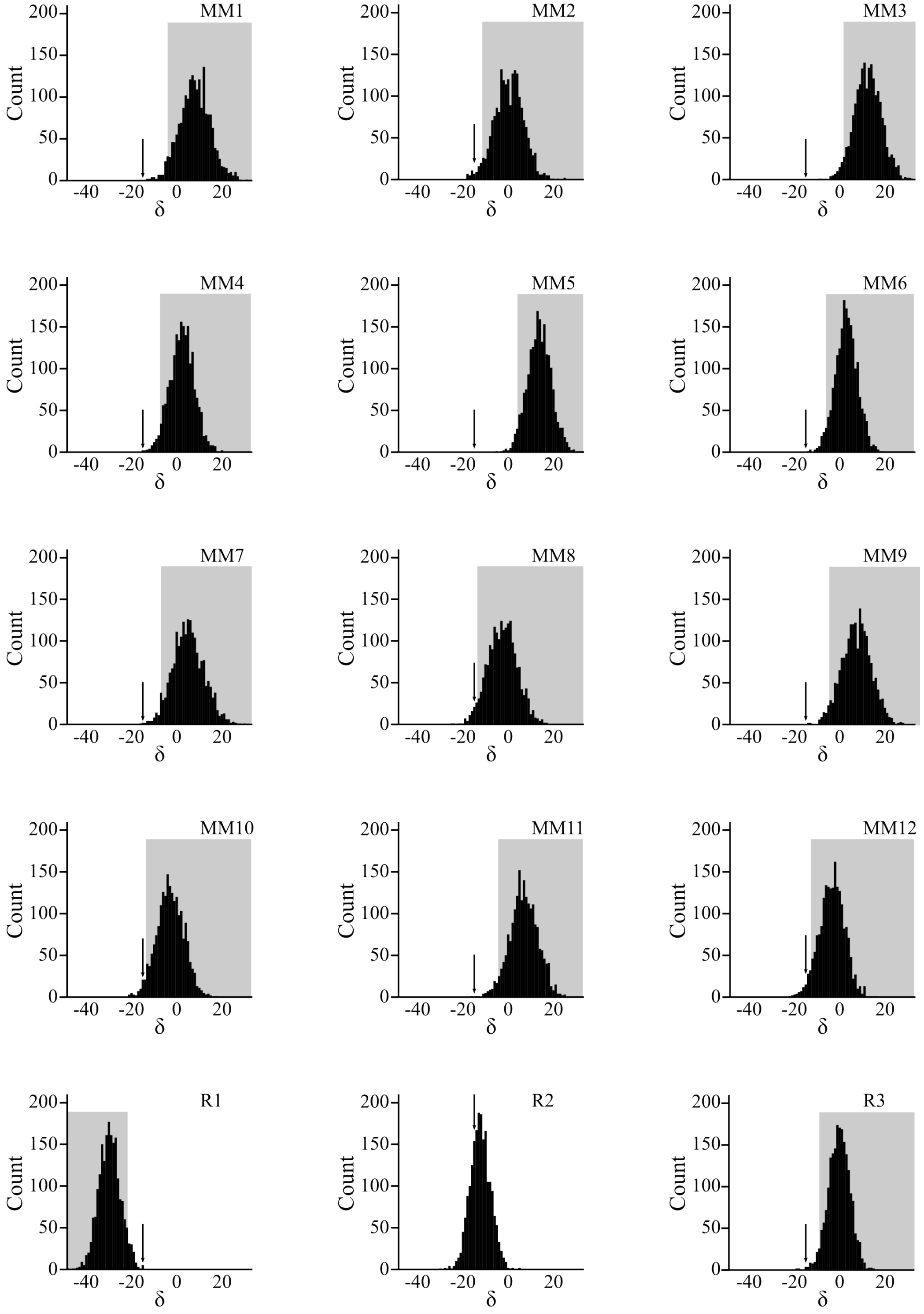

Supplement: Additional file 5 — Simulated distributions of the test statistic δ. Frequency distribution for the test statistic δ simulated in 15 models of population structure. [file 1471-2148-10-239-S5.PDF]
