# Supplementary material for: Are mimics monophyletic? The necessity of phylogenetic hypothesis tests in character evolution
Source: BMC Evol Biol. 2010 Aug 3;10:239. doi: 10.1186/1471-2148-10-239 (PMC3020633; doi:10.1186/1471-2148-10-239)

Additional File 6 for Oliver & Prudic, “Are mimics monophyletic? The necessity of phylogenetic hypothesis tests in character evolution.”

Simulated distributions of the minimum number of deep coalescences. Gene trees from 'MM' models were fit to a species tree with *L. a. astyanax* sister to *L. a. arizonensis*; gene trees from 'R' models were fit to a species tree with *L. a. astyanax* sister to *L. a. arthemis*. Arrows indicate observed value; shaded area represents the upper 95% distribution in unsupported models. Model details are found in table 2.

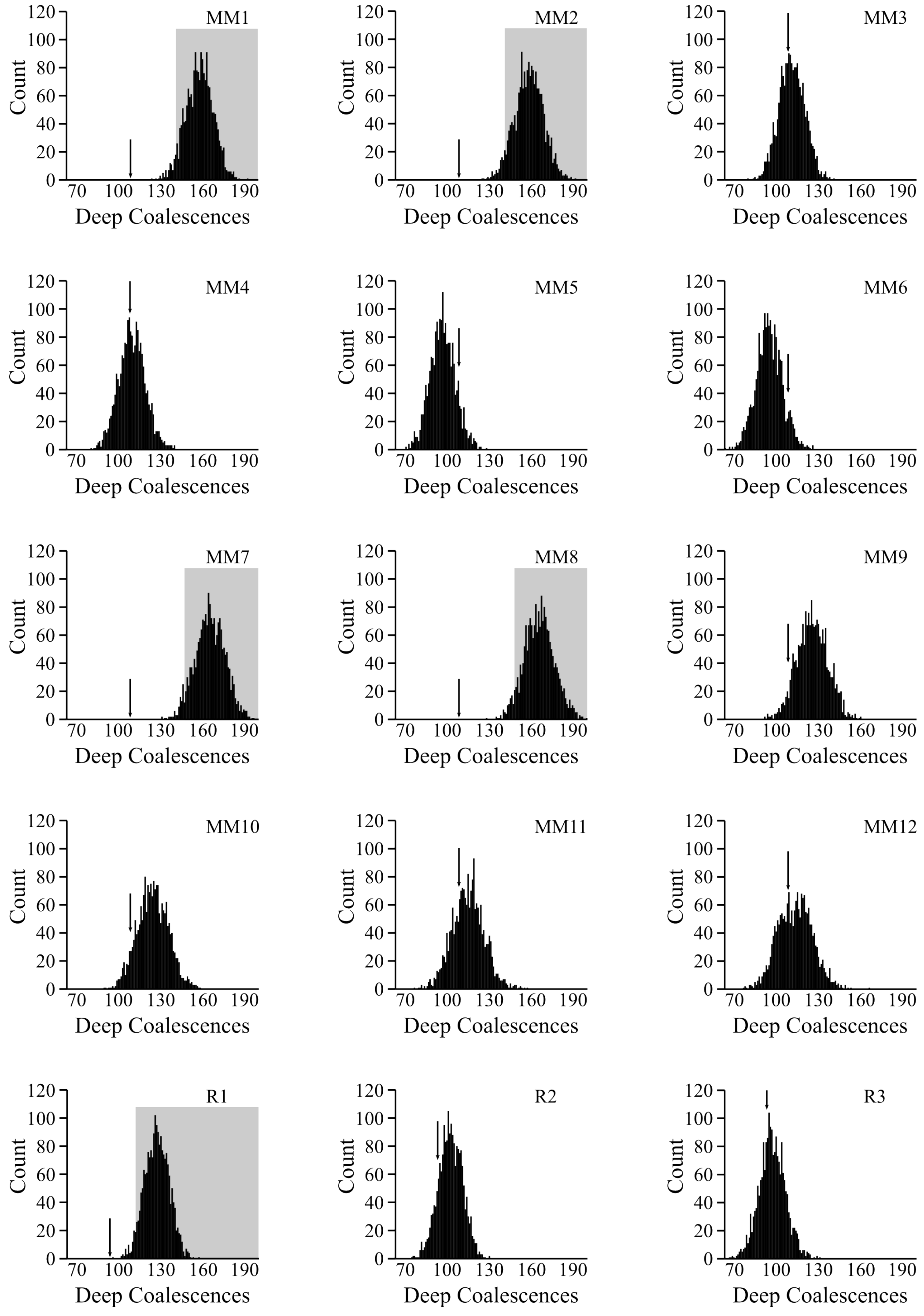

Supplement: Additional file 6 — Simulated distributions of the minimum number of deep coalescences. Frequency distribution for the number of deep coalescences simulated in 15 models of population structure. [file 1471-2148-10-239-S6.PDF]
